# Are women and men well informed about fertility? Childbearing intentions, fertility knowledge and information-gathering sources in Portugal

**DOI:** 10.1186/s12978-017-0352-z

**Published:** 2017-08-04

**Authors:** Teresa Almeida-Santos, Cláudia Melo, Ana Macedo, Mariana Moura-Ramos

**Affiliations:** 1 Faculty of Medicine, University of Coimbra, Rua Larga, 3004-504 Coimbra, Portugal; 2 Centro Hospitalar Universitário de Coimbra, Av. Bissaya Barreto e Praceta Prof. Mota Pinto, 3000-075 Coimbra, Portugal; 3 CINEICC - Cognitive and Behavioural Centre for Research and Intervention, Faculty of Psychology and Educational Sciences, University of Coimbra, Rua do Colégio Novo, 3001-802 Coimbra, Portugal; 4 KeyPoint, Edifício Miraflores Premium, Al. Fernão Lopes, n°16, 6° Andar, 1495-190 Miraflores, Portugal

**Keywords:** Fertility knowledge, Childbearing intentions, Information, Fertility protection

## Abstract

**Background:**

The postponement of parenthood may increase the number of couples experiencing infertility and prolonged time to pregnancy. Previous research has revealed that childless people are not well informed regarding fertility, which may threat their childbearing intentions. This study aimed to examine fertility knowledge and childbearing intentions held by Portuguese people and their use and perceived usefulness of information sources on fertility.

**Methods:**

Participants were recruited using a random-route domiciliary approach. A total of 2404 individuals aged 18–45 were asked to complete a structured questionnaire evaluating socio-demographic characteristics, childbearing intentions, fertility knowledge and information-gathering sources regarding fertility.

**Results:**

In total, 95.5% of the participants indicated the desire to have children in the future, and 61.7% reported that having children would contribute to life satisfaction. Most of the participants expressed the desire to have two children in the future. The discrepancy between the numbers of planned and desired children was higher in men, in participants with lower education levels, in professionally active participants and in the unemployed participants. Relationship stability seemed to be more important in influencing childbearing decisions than financial stability or family support.

Participants’ knowledge regarding fertility was poor. Women, the participants who were older than 25, the participants with longer education and the participants with higher income exhibited the greatest levels of knowledge of fertility, although this knowledge was only slightly enhanced in these subgroups. Also, the participants overestimated both the chances of spontaneous pregnancy and the success rates of assisted reproduction techniques. Finally, the results revealed that websites were the main information sources used by the participants and only 18.0% of the participants had previously discussed fertility issues with their doctors.

**Conclusions:**

Although Portuguese men and women reported the desire to have children in the future, their knowledge regarding fertility and infertility risk was poor. In addition, participants used more general sources of information, such as website, but not specialized sources, such as their doctors. There is a real need to work with general practitioners to empower them to provide adequate fertility information to every childless patient.

## Plain English Summary

The postponement of parenthood may increase the number of couples experiencing infertility and prolonged time to pregnancy. In this study, we aimed to examine fertility knowledge and childbearing intentions held by Portuguese people and their use and perceived usefulness of information sources on fertility. Participants were 2404 individuals aged 18–45 who were asked to complete a structured questionnaire evaluating socio-demographic characteristics, childbearing intentions, fertility knowledge and information-gathering sources regarding fertility. In total, 95.5% of the participants indicated the desire to have children in the future, and 61.7% reported that having children would contribute to life satisfaction. Most of the participants expressed the desire to have two children in the future, but we found a discrepancy between the number of desired and planned children. This discrepancy was higher in men, in participants with lower education levels, in professionally active participants and in the unemployed participants. We also found that participants’ knowledge regarding fertility was poor. Women, the participants who were older than 25, the participants with longer education and the participants with higher income exhibited the greatest levels of knowledge of fertility, although this knowledge was only slightly enhanced in these subgroups. In addition, participants used more general sources of information, such as website, but not specialized sources, such as their doctors. Our results confirmed that there is a real need to work with general practitioners to empower them to provide adequate fertility information to every childless patient.

## Background

For several decades, people across Europe have been postponing parenthood. As a consequence, an increasing number of couples are experiencing infertility and prolonged time to pregnancy. Assisted reproductive technology (ART) cannot fully compensate for the loss of fecundity that is linked to greater maternal age.

Fertility knowledge in the general population is poor. The evidence indicates that people are unaware of the biological aspects of conception and often overestimate the chances of achieving pregnancy at the time of ovulation [1]. People are also generally not well informed of when women are most fertile and lack understanding of the decline that occurs in female fertility after the age of 34 years [2]. Knowledge about the specific risk factors associated with decreased fertility is limited (e.g., sexually transmitted infections, smoking, alcohol consumption) [3] and erroneous with regard to factors that have no impact on fertility potential. It has been reported that women of reproductive age have insufficient fertility knowledge and awareness, with the primary areas of limited awareness being reproductive lifespan and assisted reproduction [4, 5].

Women have an acceptable understanding concerning age-associated declines in fertility but an overestimated belief in the success of assisted human reproductive treatments. Previous studies of Swedish [6], Finnish [4], American [7] British [3], Israeli [8] and Canadian [9, 10] university students have reported gaps in awareness of female infertility risk factors and an overestimation of the fertility of women in their late thirties. Studies from Sweden and Canada have shown that the understanding of female fertility among university students is deficient, especially the understanding of when female fertility declines and how this affects the chances of achieving pregnancy [1, 2, 11].

A survey that addressed some of these issues by sampling a nationally representative group of female Canadians indicated that women were only moderately knowledgeable about fertility [9].

Several studies have identified a substantial need to inform health professionals and the general public about the increased reproductive risks that are associated with increased maternal age. Provision of this information is essential to enable those who desire to become parents to make informed decisions regarding when to start having children [12]. However, the interplay between the biological, psychological, relational, and social dimensions of fertility decision-making and the realization of fertility intentions are complex processes.

Most young people in Europe want to have two or more children, with an average of 2.2 children. The mean difference between the intended and realized family size is called the fertility gap and was estimated to be 0.34 children per woman in the European Union in 2006, ranging from 0.25 in Germany and Austria to 0.71 in Northern Europe [13]. The fertility gap is generally related to personal or socio-economic factors that delay the start of childbearing. Such factors may include not having met a suitable partner, being in a broken relationship, having financial constraints, and or having competing educational, professional or personal ambitions [14]. Consequently, many couples experience tension between the desire to have children and their reasons for delaying childbearing.

Regardless of the trend of delaying childbearing, both women and men consider the late twenties the ideal time for having children. A study of a Canadian population conducted by Daniluk and Koert [9] showed that men believe the ideal age for having a first child is 29.5 years, although most do not expect to become parents before the age of 36.8 years. Women indicated that 26.8 years is the ideal age for having a first child, but most do not expect to have their first child before the age of 32.4 years [9].

Sub-optimal fertility behaviour may be influenced by attitudes towards fertility medical consultation and treatments, especially if it prevents people from seeking help. To date, most studies of fertility have focused on university students, and used online data collection methods. This focus on highly educated and well informed participants threatens the generalizability of their conclusions to the childbearing population aged between 25 and 40 years old or to less educated people. To expand current knowledge, the primary aims of the present study were to examine fertility knowledge and intentions in men and women not trying to conceive and to ascertain whether these varied across gender and contextual factors and to examine which sources of information are used to gather information about fertility. To achieve these goals, we surveyed 2400 childless individuals ranging in age from 18 to 45 years old, using a national representative sample and face-to-face data collection method to overcome limitations of the previous studies reported above. Based on the research reviewed above, we hypothesized that women with higher educational levels, those who previously engaged in medical consultations and those with higher socio-economic status would have better fertility knowledge and more favourable beliefs about treatment than other populations.

## Methods

### Procedures

This study was submitted to the Portuguese Data Protection Ethics Commission. The study used a cross-sectional design to evaluate a nationally representative sample, which was calculated separately for women and men stratified by region and age group. The final sample size was calculated using a 2:1 female–male ratio and assuming e a 2.5% error margin for women and a 3.5% error margin for men, for a 95% confidence interval (assuming the female population as the primary objective). Data were collected between August and December 2014 using a random-route domiciliary approach., i.e. for each randomly-chosen sampling points, interviewers were assigned with a starting location and provided with instructions on the random-route procedures and the selection of respondents, and the routes ended when the predefined number of respondents was achieved. This approach was considered once the probability of the selected respondents is unknown [15]. Diaz de Rada and Martínez Martín compared random-route sampling with two probabilities samples and reported that the random-route samples provide a better degree of representativeness [16]. The data collection period spanned Monday to Sunday from 10:00 to 21:00. Data were collected using a structured questionnaire, which was administered by team members trained in data collection, in ethical and data privacy, and in technical issues specific to this study. All participants gave oral informed consent prior to participation in the study. The refusal rate for answering was 20%. The questionnaire evaluated socio-demographic characteristics, childbearing intentions, fertility knowledge and information-gathering sources regarding fertility.

### Participants

The study sample included 2404 childless Portuguese women (*n* = 1596; 66.4%) and men (*n* = 808, 33.6%) aged between 18 and 45 years old and living in Continental Portugal. Individuals undergoing fertility treatments at the time of data collection were excluded.

### Measures

The questionnaire used in the study covered three main topics:

#### Childbearing intentions

This section assessed the participants’ childbearing intentions regarding 1) the numbers of desired and intended children, 2) the ideal age to have a first and last child, 3) the intended age to have a first child, 4) the importance of parenthood in life satisfaction, and 5) the experienced motives for and barriers toward parenthood. The importance of parenthood in life satisfaction was assessed using a Likert-type scale that ranged from 1 (not important) to 5 (very important). Motives for parenthood were assessed using a Likert-type scale that ranged from 1 (not valued) to 5 (highly valued). The following 4 motives were assessed: socioeconomic values (e.g., social pressure and social value), personal fulfilment (e.g., to provide meaning to life), continuity (e.g., to assure familial continuity), and marital relationship (e.g., to strengthen a marital relationship). Barriers/facilitating factors for childbearing were assessed by asking what factors (e.g., economic stability, martial relationship stability and family support) would be essential for deciding to have children in the future. Responses were provided using a Likert-type scale that ranged from 1 (not important) to 5 (very important).

#### Knowledge of fertility

This section consisted of 24 statements (e.g., “A 40-year-old woman has similar odds to a 30-year-old woman for achieving pregnancy”, “If a woman is obese, this may affect odds of achieving a pregnancy”), and the participants were asked to score each as true or false. Correct answers were scored as 1, and incorrect answers were scored as 0. If a participant failed to provide an answer, then the statement was coded as incorrect (0). Knowledge of fertility and of infertility risk factors was calculated by summing all the answers.

#### Information gathering about fertility issues

The participants were asked about what sources they used to gather information about fertility (each source was coded 0 for not used and 1 for used) and how useful they considered them to be. The list of sources included media, family and friends, and physicians. The list was also evaluated using a Likert-type scale that ranged from 1 (not useful) to 5 (very useful). In addition, specific questions were asked regarding information provided by general practitioners.

### Data analysis

Data were analysed using IBM SPSS, version 20.0 (IBM Corporation, Armonk, NY, USA). Power calculations demonstrated that the achieved sample size was sufficient to detect small effects for analysis with a power of .90 (G*Power). The significance level was set at 0.05. Comparisons between subgroups of participants were conducted using analysis of variance (ANOVA) with Bonferroni post hoc analysis. ANOVA for repeated measures was used to evaluate discrepancies between behavioural intentions and desires and planned behaviour.

## Results

### Description of participants

The main socio-demographic characteristics of the participants are reported in Table 1.

**Table 1:** Participants’ characteristics

	Women (*n* = 1596; 66.4%)	Men (*n* = 808; 33.6%)	*p*
Age (mean ± SD)	32.9 ± 7.9	32.3 ± 8.0	.796
Years of education			
Basic school (9 years)	305 (20.1%)	141 (18.5%)	.407
Secondary (12 years)	673 (44.5%)	361 (47.3%)	
University	536 (35.4%)	262 (34.3%)	
Work status			
Student	260 (16.3%)	138 (17.1%)	.001
Employed	1087 (68.2%)	587 (72.9%)	
Not employed	247 (15.5%)	80 (9.9%)	
Income per month			
No income	407 (25.8%)	183 (22.8%)	< .001
< 750€	555 (35.1%)	140 (17.5%)	
750–1500€	463 (29.3%)	367 (45.8%)	
> 1501€	83 (5.2%)	57 (7.1%)	
Not provided	71 (6.7%)	54 (4.5%)	
Currently involved in a relationship	1020 (63.9%)	537 (66.5%)	.216

The study participants included both men and women around 32 years old. Most participants had completed secondary education and were employed. The majority of the participants were not married but were currently in a relationship. The large majority of the participants identified as heterosexual (98.9%). We excluded participants who described themselves as homosexual because, at the time of data collection, the use of assisted reproduction techniques in Portugal was exclusive to heterosexual couples who were married or had been cohabiting for longer than two years. As such, information on fertility and childbearing intentions may have been bounded to this condition.

### Childbearing intentions

Approximately 95.5% of the participants in the study reported the desire to have children in the future, and 61.7% reported that having children in the future would contribute positively to their life satisfaction (M = 3.67, SD = .98). Differences were found between men and women (M = 3.53, SD = 1.03 and M = 3.74, SD = .95, respectively), but they were relatively small (Cohen’s d = 0.2).

#### Desired and planned children in the future

**Fig. 1:**
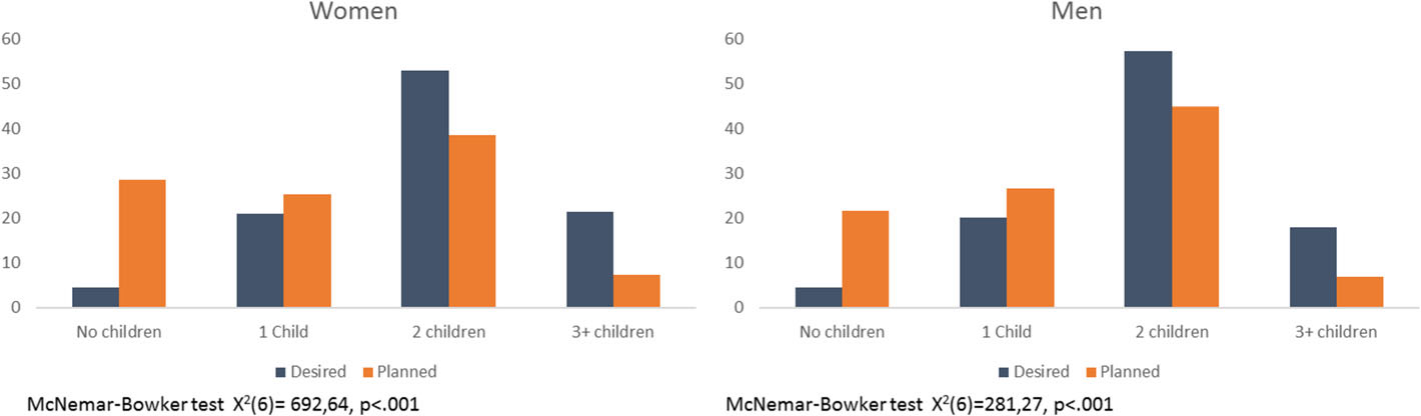
Discrepancy between the number of desired and number of planned children. Values are percentage of participants in each category

Approximately 36 (4.6%) men and 70 (4.4%) women reported they did not want to have children in the future. Most participants reported wanting to have two children in the future (*N* = 1282, 53.8%). Men and women did not differ regarding the number of children they desired (*X* ^*2*^_*3*_ *=* 5.68*, p* = .128). Our results also showed that the participants planned fewer children (*M* = 1.29, *SD* = 0.95) than they desired to have (*M* = 1.94, *SD* = .85; *t* = 35.06, *p* < 0.001) (see Fig. 1). This discrepancy was significantly higher for men (*F*_1,2345_ = 15.85, *p* < 0.001), for participants with lower education levels (*F*_2,2221_ = 167.16, *p* < 0.001), and for the participants who were professionally active or unemployed compared to those who were students (*F*_2,2339_ = 68.73, *p* < 0.001). The discrepancy between the number of desired and number of planned children was also significant between age groups (*F*_2,2344_ = 326.77, *p* < .0.001). Participants up to 25 years old reported the smallest discrepancy (M = .17, SD = 0.68), while the oldest group (above 35 years old) reported the highest discrepancy (M = 1.13, SD = 0.98).

#### Ideal and planned age to have children

The participants considered 29.20 years as the ideal age to have a first child (SD = 2.8), but most planned on having their first child by the age of 32.84 years (SD = 4.80). This discrepancy was statistically significant (F_1,1706_ = 1243.31, *p* < 0.001) and was not different between men and women (F_1,1705_ = .68, *p* = .409).

Differences between the ideal age and the planned age for a first child were found according to all three education levels of the groups (F_2,2494_ = 14,83, *p* < 0.001), with the participants having 9 years or less of education showing the steepest discrepancy (M = 5.18 SD = 4.83) compared to those who had completed secondary education (*M* = 3.14; *SD* = 4.10) or university education (*M* = 3.69; *SD* = 3.94). This discrepancy was also significantly different across the four levels of income (F_3,1242_ = 18,54, *p* < 0.001) and increased in significance as income level increased. Finally, the discrepancy was also significant according to professional status (F_2,1700_ = 147.97, *p* < 0.001), with students reporting the lowest gap (M = 0.64, SD = 1.80) and professionally active individuals reporting the highest gap (M = 4.68, SD = 4.08). These results are presented in Table 2.

**Table 2:** Differences between ideal and planned age for childbearing

	Ideal age	Planned age	Difference	*p*
Gender				
Men	30.11 ± 3.05	33.86 ± 4.91	−3.76	< .001
Women	28.70 ± 2.57	32.28 ± 4.64	−3.58	< .001
Age groups				
18–25	28.28 ± 2.62	28.92 ± 2.88	−0.64	< .001
26.35	29.30 ± 2.59	32.38 ± 2.81	−3.08	< .001
> 36	30.10 ± 2.83	38.72 ± 4.11	−8.62	< .001
Education				
9 years	28.40 ± 2.84	33.58 ± 5.20	−5.18	< .001
12	28.80 ± 2.56	31.94 ± 6.67	−3.14	< .001
University	29.79 ± 2.94	33.48 ± 4.34	−3.69	< .001
Professional status				
Student	28.65 ± 2.86	29.29 ± 3.07	−0.64	< .001
Unemployed	29.36 ± 2.84	34..03 ± 4.73	−3.58	< .001
Active	29.36 ± 2.65	32.95 ± 4.60	−4.68	< .001

#### Motives for parenthood and factors affecting childbearing decisions

**Fig. 2:**
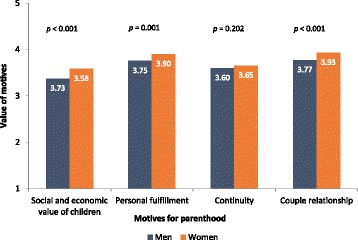
Motives for parenthood for men and women. Motives were defined as Social and economic value of children, such as social importance of parenthood, economical support for parents in the future; Personal fulfilment, as contributing for meaning of own life; Continuity, such as importance of genetic continuity and family lineage. Participants rated each motive from 1 to 5. T tests for independent samples were conducted to compare value of motives between men and women

**Fig. 3:**
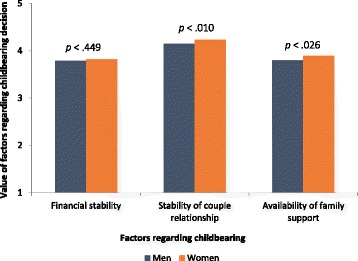
Factors valued by the participants regarding childbearing decision among men and women. Participants rated each motive from 1 to 5. T tests for independent samples were conducted to compare value of motives between men and women

Figures 2 and 3 display, respectively, the most important motives for parenthood and the main factors affecting childbearing decisions.

For both men and women, the most valued motives for parenthood were related to personal fulfilment (*M* = 3.85, *SD* = 0.98) and to the couple’s relationship (*M* = 3.88, *SD* = 0.95). Similarly, the stability of a couple’s relationship was the most important factor affecting childbearing decisions (*M* = 4.21, *SD* = 0.79) compared to financial stability (*M* = 3.80, *SD* = 0.98) and the availability of family support (*M* = 3.86, *SD* = 0.94).

### Knowledge of fertility and assisted reproduction techniques

#### Knowledge of fertility and infertility risks

**Fig. 4:**
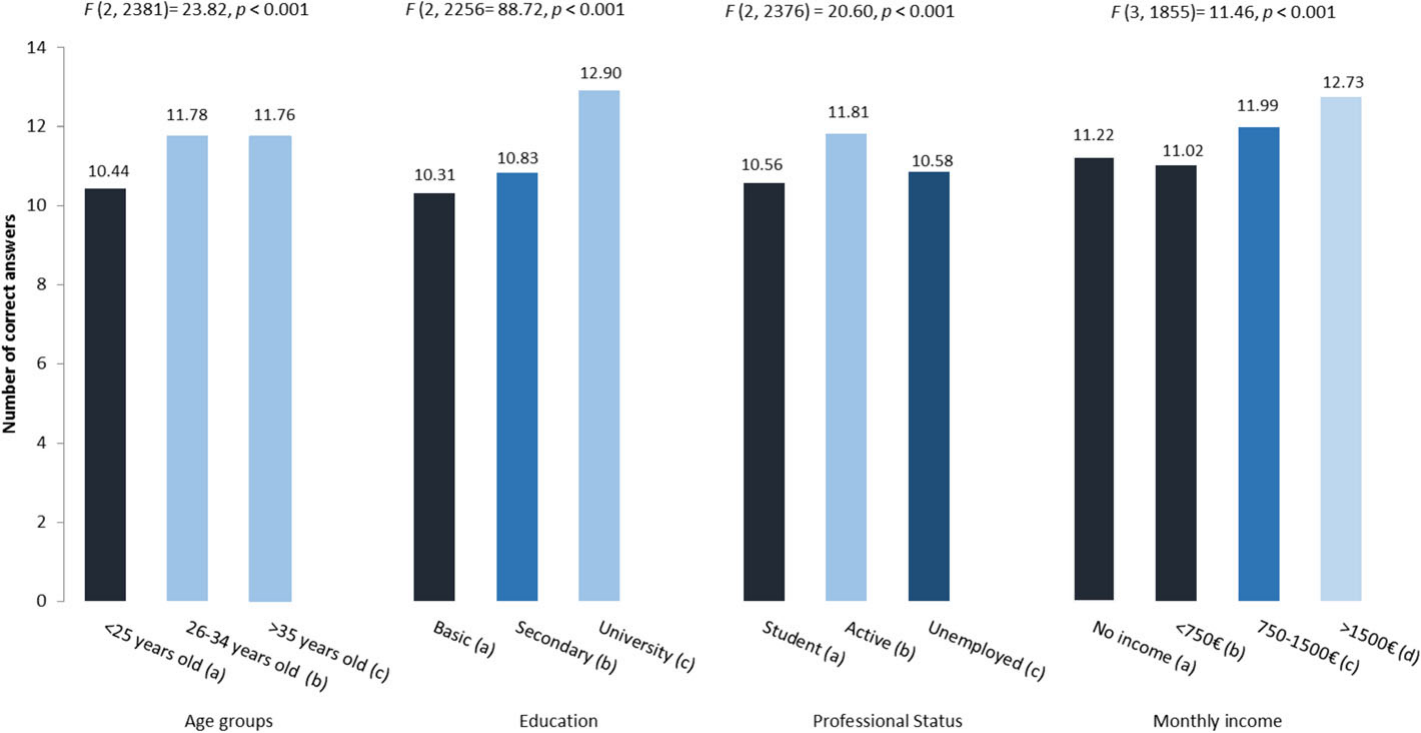
Number of correct answers per subgroup according to age, education, professional status and monthly income. *Group comparisons:* Age groups: a < b, (*p <* 0.001); a < c, (*p* < 0.001); b = c, (*p* = 0.999). Education: a < b (*p* < 0.049), a < c (*p* < 0.001); b < c (*p* = 0.999). Professional status: a < b (*p* < 0.001), a < c (*p* < 0.999); b > c (*p* < 0.001). Monthly income: a = b (*p* = 0.990), a = c (*p* < 0.082); a < d (*p* = 0.004); b < c (*p* < 0.001); b < d (*p* < 0.001); c = d (*p* = .252)

A set of 24 statements referring to human fertility knowledge and infertility risk factors was presented to the participants, who were asked to score each statement as correct or incorrect. The results revealed that the participants generally had poor knowledge of fertility, as they only scored approximately half of the statements correctly (*M* = 11.50, *SD* = 3.97). Women scored more statements correctly than men (11.8 vs. 10.8, *t* = −5.94, *p* < 0.001). Significant differences in the number of correct answers were found according to age group (F_2,2381_ = 23.82, *p* < 0.001), education level (F_2,2256_ = 88.72, *p* < 0.001), professional status (F_2,2376_ = 20.60, *p* < 0.001), and income level (F_3,1855_ = 11.46, *p* < 0.001), although in all cases the differences were small. Figure 4 reports the number of correct answers given per group of participants.

### Knowledge regarding chances of achieving pregnancy spontaneously and after undergoing one cycle of assisted reproduction

The participants tended to overestimate their changes of achieving pregnancy, and no differences were found between men and women (*χ*2 = 3.71, *p* = .295). Approximately 68.7% of the participants reported that a healthy couple has a greater than 50% chance of becoming pregnant each month, and only 9.3% reported odds of achieving pregnancy per month of less than 25%.

The participants also overestimated the success rates of ART. Approximately 45.5% of the participants reported that more than half of couples would achieve pregnancy after the first ART cycle.

### Gathering information on fertility and infertility risks

#### Use and perceived usefulness of sources of information on fertility and infertility risks

**Fig. 5:**
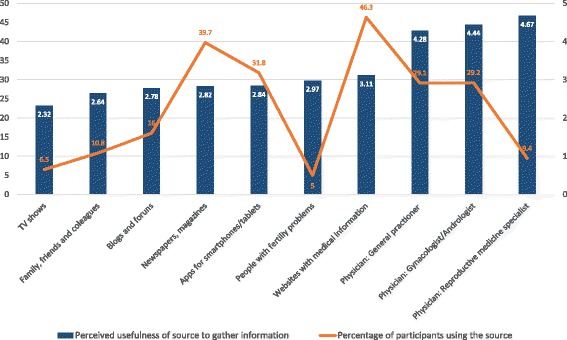
Information sources typically used by the participants and their perceived usefulness regarding fertility and reproductive health. The blue columns refer to the perceived usefulness of each source of information for gathering knowledge on fertility. The orange line refers to the percentage of participants who actually used each listed information source to gather knowledge on fertility

The participants were assessed regarding the sources of information they typically used and on the perceived usefulness of each source. Figure 5 displays the results for these two categories.

The results showed that few of the participants had used the sources of information that they perceived as the most useful, such as doctors. The main information sources that were used were websites containing medical information and newspapers and magazines, and these were perceived as only moderately useful. Medical doctors, who were considered very useful sources of information, were used by less than 30% of the participants.

#### Discussion of fertility issues with doctors

Only 18.0% of the participants reported that they had discussed fertility issues during appointments with medical doctors. Of these, 34.6% participants reported discussing these issues with their general practitioner, and 48.1% reported discussing them with their gynaecologist or fertility doctor. When fertility issues were discussed during medical appointments, the participants reported that the topic was discussed often (33.3%), sometimes (46.6%) or almost never (12.6%). In most situations, the topic was raised by the patients (71.5%) compared to by a doctor (28.5%).

## Discussion

The present study described childbearing intentions of, knowledge of fertility possessed by and information gathering sources used by a childless Portuguese population. To the best of our knowledge, this is the only large-scale study that has assessed fertility knowledge in a representative national sample using a door-to-door recruitment procedure, overcoming the limitations of previous studies that collected data using online methods and mainly recruited university students, threatening the generalizability of this conclusions to the general population.

Concordant with previous studies, in the current work, the majority of participants planned to have children in the future, although there was a discrepancy between their expressed desires and their plans. The majority of participants in our sample wanted to have two children, although a significant proportion did not make plans to accomplish this target. This discrepancy was more pronounced in the older population, probably because of the reduced time left to this population for the completion of reproductive plans. Additionally, conditions such as lack of relationship stability, financial stability or family support were associated with higher discrepancies. The postponement of parenthood relative to what is desired may also contribute to the perception that the ideal family size will not be reachable, even though our participants were not aware that fertility declines as women age. Other studies have also shown that there is a considerable difference between intended and realized family size; this fertility gap may be up to half a child among European countries [1, 12].

Our results also highlighted that, in general, both men and women are poorly informed about fertility and infertility risk factors. Indeed, the participants scored less than half of the statements on the questionnaire correctly, which is in accordance with other findings, although the use of different items to evaluate fertility knowledge prevents comparisons to published results.

Our results suggest that education is positively correlated with fertility knowledge, in accordance with other studies [11, 17]. However, in our study, neither the women nor the men were sufficiently aware of the age-related decline that occurs in female fecundity, and both the women and the men had optimistic perceptions of women’s chances of becoming pregnant. These results are in agreement with previous studies, including one that evaluated university students in Sweden [1]. Our sample included people with low education levels, unlike previous studies, which have predominantly included university students or people attending fertility clinics, who tend to be more informed on these subjects. The low fertility knowledge of this less educated population may reflect a general health illiteracy and emphasizes the need to develop strategies to increase the information available to such populations.

Fertility knowledge also varied in terms of socio-demographic variables, with those who had higher income, those who worked, those who were older and those who had greater education being better informed. The lower fertility knowledge exhibited by the participants who were younger than 25 may have reflected the inclusion of undergraduate students, as this population is generally not making childbearing decisions or seeking fertility information.

Finally, the lack of knowledge exhibited by the participants may have resulted from the sources of information that Portuguese men and women tend to use. Indeed, although the participants considered general practitioners and fertility specialists the most useful and trustworthy sources of information, they tended to use other sources instead. In addition, we also verified that general practitioners seldom took the initiative to provide information on fertility and infertility risks.

A major strength of the present work was the method used for sample recruitment. A door-to-door method was employed, which allowed the collection of data from a representative Portuguese population. This method enabled us to recruit participants regardless of their ability to access the internet and regardless of whether they were students, which are major limitations of previous studies on this topic [1, 9, 10]. As a consequence, participants with several levels of education, incomes and professional statuses were included in the study. Another important strength of our study was the inclusion of large numbers of both men and women. Indeed, most other studies have focused predominantly on women and have not evaluated men’s knowledge on fertility. This is linked to another important strength of the study, which was the assessment of the study group’s knowledge of specific risk factors for male infertility and the evaluation of gender differences with regard to knowledge and the sources of information used. Finally, the evaluation of the main sources of information used by men and women was another important strength, as it may contribute to the development of specific strategies to increase fertility knowledge in populations of reproductive age.

Albeit these strengths, it is important to note that the recruitment strategy that was used may have an influence in study results. Indeed, it has been documented that random route strategy may have some bias in the representation of some participants’ variables [16], namely by overrecruiting participants from subgroups of the population (in general, younger or older, unemployed) [18]. However, we believe that the inclusion criteria (age range, childlessness) and the recruitment during a large period of the day and during both weekdays and weekends, has highly reduced this bias.

### Implications for public health decisions and information campaigns

As the provision of information regarding fertility is essential for helping those who wish to become parents to make qualified decisions on when to start having children [12], there is a real need to work with general practitioners, particularly those working in family planning, to empower them to provide adequate fertility information to every childless patient.

## Conclusion

Men and women have little information on the factors that affect their fertility, namely on modifiable behavioural risk factors. This lack of information may not only prevent individuals from correcting their behaviour in order to protect their fertility but also from requiring additional information on this topic from the most relevant and trustworthy sources of information. The provision of information on fertility is of uttermost importance in order to promote informed decisions on planning childbearing.

## Abbreviations

ANOVA: Analysis of variance
ART: Assisted reproductive technology
IBM SPSS: Statistical Package for Social Sciences
